# A Collaborative Framework Based on an Improved Adaptive Cubature Kalman Filter for Multi-Anomaly Mitigation in Bridge Temperature Monitoring Data

**DOI:** 10.3390/s26165061

**Published:** 2026-08-10

**Authors:** Benkun Tan, Zhixue Hu, Shengtao Xiang, Da Wang, Jialin Shi, Zujun Zhang, Fanghuai Chen, Guoliang Zeng

**Affiliations:** 1 School of Civil Engineering and Architecture, Hunan University of Arts and Science, Changde 415000, China; tanbenkun@huas.edu.cn (B.T.); zhixuehw@yeah.net (Z.H.); zhangzujun@huas.edu.cn (Z.Z.); bridgezgl3403@huas.edu.cn (G.Z.); 2 Changsha County Housing and Urban-Rural Development Bureau, Changsha 410004, China; 3 School of Materials and Energy, Central South University of Forestry & Technology, Changsha 410004, China; 20230100079@csuft.edu.cn; 4 School of Civil and Environmental Engineering, Hunan University of Technology, Zhuzhou 410114, China; chenfanghuai@hut.edu.cn

**Keywords:** structural health monitoring, bridge temperature monitoring, data cleaning, cubature Kalman filter, multi-anomaly mitigation

## Abstract

Long-term bridge temperature monitoring data are often affected by random noise, outliers, and sensor drift, which may reduce the reliability of structural thermal-response analysis. This study proposes a collaborative framework based on an improved adaptive cubature Kalman filter (IACKF) for multi-anomaly mitigation. First, the process- and observation-noise covariance matrices are updated online using innovation and residual statistics to suppress time-varying noise. Second, a dual-Gaussian contaminated observation model is incorporated to develop an outlier-resistant improved adaptive cubature Kalman filter (OR-IACKF) for isolated and patch-type outliers. Third, an improved particle swarm optimization–backpropagation–IACKF (IPSO-BP-IACKF) scheme is used to predict a drift-free reference from adjacent monitoring points and recursively estimate sensor drift. The framework is evaluated using simulated data, constant-temperature chamber measurements, field-monitored bridge data, and a jointly contaminated dataset. The results indicate that the staged framework improves the mitigation of different anomaly types while maintaining relatively stable performance under different parameter settings. The proposed method provides a practical data-processing approach for long-term bridge structural health monitoring.

## 1. Introduction

Bridge structural health monitoring (SHM) systems continuously collect multi-source data to evaluate structural behavior, service performance, and long-term safety [1,2,3]. Among these measurements, temperature data are essential for evaluating structural thermal behavior, temperature actions, and temperature-induced responses [1]. However, long-term measurements may be affected by random noise, outliers, and sensor drift because of limited sensor accuracy, transmission disturbances, environmental interference, temporary faults, and sensor aging [4,5]. Random noise generally appears as high-frequency fluctuations, outliers occur as isolated spikes or short abnormal segments, whereas drift produces a slowly varying baseline deviation. The coexistence of these anomalies may reduce the reliability of subsequent temperature-effect analysis.

Various methods have been developed to improve monitoring-data quality. Wavelet transform, time–frequency analysis, conventional smoothing, and Kalman-filter-based methods are commonly used for noise reduction [6,7,8,9,10]. Statistical detection, machine-learning classification, model-based prediction, and robust estimation have been applied to abnormal observations [11,12,13,14,15,16,17]. Sensor drift is generally corrected using reference sensors, physical models, Gaussian-process regression, or the spatiotemporal correlations among neighboring sensors [18,19,20,21,22,23]. Recent studies have explored edge intelligence and lightweight machine-learning models for decentralized SHM data processing, enabling onboard anomaly classification and sensor-fault diagnosis while reducing data-transmission loads and delays associated with centralized cloud-based analytics [24,25]. Nevertheless, many existing studies mainly focus on individual anomaly types, and the coordinated processing of noise, outliers, and drift under jointly contaminated conditions remains insufficiently investigated [26,27]. Existing multi-anomaly processing methods may generally be divided into one-step joint estimation and sequential processing. One-step methods incorporate several uncertainty sources into a unified robust or probabilistic model, whereas sequential methods use separate modules for different anomaly types. The former provides a compact formulation, but distinguishing high-frequency noise, abrupt outliers, and slowly varying drift within a single model may be difficult. The latter offers clearer processing logic, although the interactions among different anomaly types are not always explicitly considered. For instance, noise may affect outlier identification, outliers may influence drift-reference prediction, and drift may alter the local data baseline. Based on these considerations, this study develops a collaborative improved adaptive cubature Kalman filter (IACKF) framework. First, the process- and observation-noise covariance matrices are adaptively updated using innovation and residual statistics for noise reduction. Second, a dual-Gaussian contaminated observation model is introduced to construct an outlier-resistant IACKF (OR-IACKF), in which observations with larger deviations are assigned lower weights. Third, an improved particle swarm optimization–backpropagation–IACKF (IPSO-BP-IACKF) scheme uses adjacent monitoring points to predict a drift-free reference and recursively estimate long-term sensor drift without an external reference sensor.

The three modules are applied in the order of denoising, outlier suppression, and drift calibration. This sequence is intended to reduce the influence of background noise on outlier processing and the influence of abnormal observations on drift-reference prediction. The proposed framework is evaluated using simulated data, constant-temperature chamber measurements, field-monitored bridge data, and a jointly contaminated dataset. Comparative tests and parameter sensitivity analyses are conducted to examine the performance of the proposed framework under different conditions. Its computational characteristics and potential for near-real-time implementation are also discussed.

## 2. Methodology

### 2.1. Collaborative Framework for Multi-Anomaly Mitigation

Bridge temperature monitoring data are commonly contaminated by random noise, outliers, and long-term sensor drift. These three types of anomalies differ substantially in both temporal characteristics and statistical behavior. Random noise is mainly manifested as high-frequency fluctuations caused by sensor precision limitations, transmission disturbances, and environmental interference. Outliers usually appear as isolated abnormal spikes or short clustered segments that deviate markedly from the underlying temperature trend [28]. By contrast, sensor drift is a slowly evolving and cumulative baseline deviation induced by sensor aging, environmental exposure, or calibration deterioration. Owing to such heterogeneous characteristics, a single data-cleaning strategy is generally insufficient for long-term bridge structural health monitoring [22].

**Figure 1:**
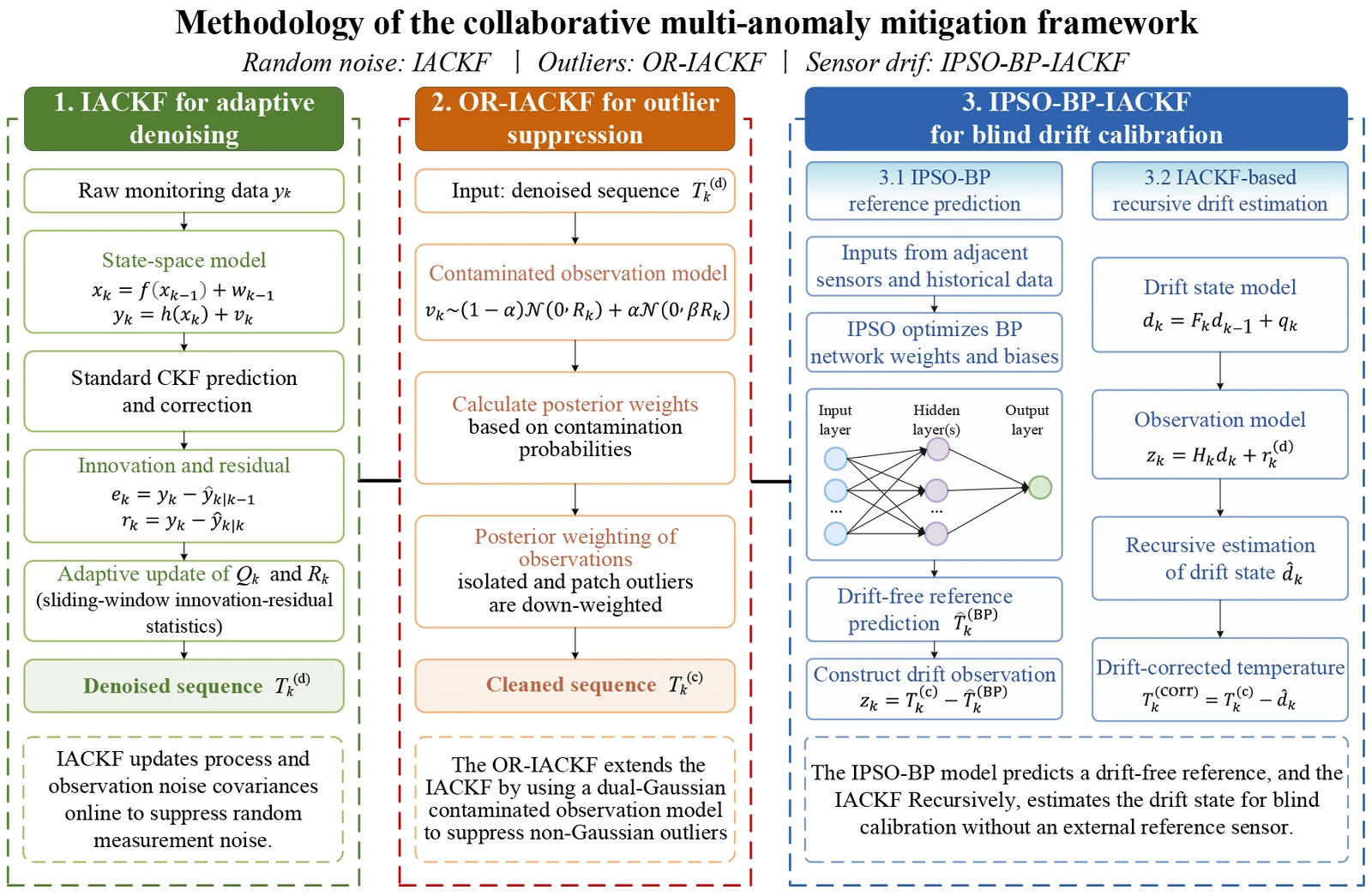
Flowchart of the proposed collaborative multi-anomaly mitigation framework.

A collaborative multi-anomaly mitigation framework is established in this study, as illustrated in Figure 1. The framework consists of three successive stages. First, an IACKF is employed to suppress random noise by adaptively updating the process and observation noise statistics during recursive estimation. Second, an OR-IACKF is developed to reduce the influence of abnormal observations through a contaminated observation model and posterior weighting mechanism. Third, an IPSO-BP-IACKF blind calibration module is constructed to correct long-term sensor drift without requiring an external reference sensor.

A staged strategy was adopted because the three anomaly types exhibit different statistical and temporal characteristics. Random noise is generally high-frequency and continuously distributed, outliers are sparse and abrupt, whereas sensor drift varies slowly over time. A single filtering model must simultaneously accommodate these conflicting characteristics, which can increase parameter coupling and cause one anomaly type to distort the estimation of another. In the proposed sequence, the IACKF first stabilizes the innovation statistics by reducing random noise, the OR-IACKF then suppresses abrupt contaminated observations, and the IPSO-BP-IACKF finally estimates the remaining low-frequency drift. This serial processing reduces cross-interference and allows each module to operate on data that better satisfy its underlying assumptions.

### 2.2. State-Space Formulation of Bridge Temperature Monitoring Data

Let *x_k_* denote the true structural temperature state at time step *k*, and let *y_k_* denote the corresponding sensor measurement. The bridge temperature monitoring process can be described by the following nonlinear discrete-time state-space model, as shown in Equations (1) and (2).

(1) $$x_{k}=f\left(x_{k-1}\right)+w_{k-1}$$

(2) $$y_{k}=h\left(x_{k}\right)+v_{k}$$

where $f\left(x_{k-1}\right)$ and $h\left(x_{k}\right)$ are the nonlinear state transition and observation functions, respectively. The term *w_k_*_−1_ represents the process noise associated with model uncertainty, whereas *v_k_* denotes the observation noise introduced by sensor and measurement disturbances. Their covariance matrices are denoted by *Q_k_* and *R_k_*, respectively.

In practical bridge monitoring, however, both *Q_k_* and *R_k_* are generally unknown and may vary with time. Moreover, the observation sequence may simultaneously contain random noise, outliers, and drift. Therefore, the raw measurement *y_k_* cannot be directly regarded as a reliable representation of the actual temperature state, and an anomaly-oriented estimation framework is required.

### 2.3. IACKF for Adaptive Denoising

#### 2.3.1. CKF-Based Nonlinear Recursive Estimation

The CKF is adopted as the basic nonlinear recursive estimation framework. At each time step, the CKF propagates a set of cubature points through the nonlinear state equation and observation equation to obtain the predicted state and the updated posterior state [29]. Compared with linearization-based approaches, the CKF provides improved nonlinear estimation capability while maintaining moderate computational cost.

Nevertheless, the denoising performance of the standard CKF depends strongly on the preset process noise covariance *Q_k_* and observation noise covariance *R_k_*. When these covariance matrices are inaccurately specified or vary with time, the filter may assign inappropriate confidence to the state model and the measurements, which can degrade estimation accuracy and stability. To overcome this limitation, an adaptive covariance updating strategy is introduced [30].

#### 2.3.2. Innovation-Residual-Based Adaptive Covariance Update

To improve robustness under time-varying noise conditions, the standard CKF is extended to an IACKF [30,31]. The key idea is to revise *Q_k_* and *R_k_* online according to the innovation and residual information generated during recursive estimation.

The innovation sequence and residual sequence are shown in Equations (3) and (4).

(3) $$e_{k}=y_{k}-{\hat{y}}_{k|k-1}$$

(4) $$r_{k}=y_{k}-{\hat{y}}_{k|k}$$

where ${\hat{y}}_{k|k-1}$ is the predicted observation and ${\hat{y}}_{k|k}$ is the updated observation estimate.

In Equations (3) and (4), *e_k_* quantifies the discrepancy between the current measurement and the model-based prediction, whereas *r_k_* characterizes the remaining discrepancy after the update step. In the IACKF, the statistical characteristics of the innovation and residual sequences are estimated within a sliding window of length *N*. Based on these windowed statistics, the observation noise covariance *R_k_* is updated adaptively, and the process noise covariance *Q_k_* is further adjusted according to the relationship between measurement inconsistency and state-prediction error. In this way, the filter can continuously adapt its confidence in both the state evolution model and the measurement sequence.

#### 2.3.3. Stability and Sliding-Window Selection

To improve numerical stability, the adaptively estimated covariance matrices are first symmetrized and then constrained by eigenvalue projection as

(5) $$Q_{k}=\Pi_{\left[\varepsilon_{Q},Q_{\max}\right]}\left(\frac{{\hat{Q}}_{k}+{\hat{Q}}_{k}^{T}}{2}\right),\;\;R_{k}=\Pi_{\left[\varepsilon_{R},R_{\max}\right]}\left(\frac{{\hat{R}}_{k}+{\hat{R}}_{k}^{T}}{2}\right),$$

where Π denotes eigenvalue projection and *ε_Q_*, *ε_R_* > 0. Therefore, *Q_k_* and *R_k_* remain positive definite. To avoid abrupt covariance variation under extreme temperature fluctuations, the updates are smoothed as

(6) $$Q_{k}=(1-\lambda_{Q})Q_{k-1}+\lambda_{Q}{\hat{Q}}_{k},\;\;R_{k}=(1-\lambda_{R})R_{k-1}+\lambda_{R}{\hat{R}}_{k},$$

where 0 < $\lambda_{Q}$, $\lambda_{R}$ < 1. These constraints preserve the positive definiteness of the covariance matrices and suppress abrupt variations in the adaptive covariance estimates, thereby improving the numerical stability of the recursive filtering process and reducing the risk of filter divergence.

The sliding-window length *N* balances statistical stability and responsiveness. The sliding-window length *N* was selected through the sensitivity analysis presented in Section 4.1.3.

#### 2.3.4. Denoised Output

After adaptive recursive estimation, the IACKF yields a denoised temperature sequence, denoted by *T_k_*^(d)^, where the superscript (d) indicates that the sequence has mainly undergone random-noise suppression. This denoised sequence is subsequently used as the input for the outlier-suppression stage shown in Figure 1.

### 2.4. OR-IACKF for Outlier Suppression

Although the IACKF can effectively reduce random noise, it remains sensitive to non-Gaussian abnormal observations [30,31,32]. In long-term bridge temperature monitoring, such observations may be caused by temporary sensor malfunction, signal transmission interference, or abrupt environmental disturbances [33], and may appear either as isolated outliers or as short abnormal clusters. These contaminated measurements may distort the innovation statistics and thereby weaken the robustness of recursive estimation.

To enhance robustness against such contamination, a dual-Gaussian contaminated observation model is introduced into the IACKF framework, yielding an OR-IACKF. The observation noise is expressed as

(7) $$v_{k}\sim (1-\alpha )N(0,R_{k})+\alpha N(0,\beta R_{k})$$

where *α* denotes the contamination ratio, representing the proportion of abnormal observations, and *β* is the covariance inflation factor used to describe the enlarged uncertainty associated with contaminated measurements.

In Equation (7), the first Gaussian component corresponds to nominal measurement noise under normal conditions, whereas the second component represents the heavy-tailed disturbance induced by outliers. Based on this contaminated observation model, posterior weighting is introduced into the update stage. Consequently, observations that remain consistent with the predicted temperature trend retain relatively large influence, whereas observations with stronger abnormality are assigned lower weights. This strategy enables both isolated and patch outliers to be adaptively down-weighted while preserving the underlying temperature variation pattern.

After the OR-IACKF stage, the cleaned temperature sequence is denoted by *T_k_*^(c)^, where the superscript (c) indicates that the sequence has undergone both denoising and outlier suppression. This cleaned sequence serves as the input for subsequent blind drift calibration.

### 2.5. IPSO-BP-IACKF for Blind Drift Calibration

#### 2.5.1. Drift-Free Reference Prediction Using IPSO-BP

Although the OR-IACKF can suppress random noise and outliers, it cannot directly eliminate long-term sensor drift. In bridge temperature monitoring, drift usually appears as a slow and cumulative baseline deviation. Unlike random noise and outliers, drift does not mainly manifest as local fluctuation or sudden abnormality, but rather as a persistent bias superposed on the true temperature evolution [20,21,22]. To address this problem, a blind drift calibration framework combining IPSO-BP prediction and IACKF-based recursive estimation is developed.

The proposed calibration framework relies on the spatiotemporal correlations among adjacent monitoring points and uses a BP neural network to establish the nonlinear relationship between neighboring measurements and the target temperature response [34]. After denoising and outlier suppression, the cleaned measurements from neighboring sensors are used as the input features of a backpropagation neural network, while the early-stage data of the target monitoring point without obvious drift are used as the training labels. After training, the network provides a drift-free reference prediction for the target sensor. The predicted drift-free temperature at time step *k* is denoted by ${\hat{T}}_{k}^{\left(\mathrm{BP}\right)}$, where the superscript (BP) indicates that the value is generated by the BP network.

To improve convergence and avoid poor local optima, an IPSO algorithm is employed to optimize the initial weights and biases of the BP network [35,36,37]. Therefore, the IPSO module in Figure 1 is used to determine the optimal network parameters, rather than to directly estimate the drift itself.

#### 2.5.2. Drift State Model

After obtaining the drift-free reference, the sensor drift is modeled as a slowly varying latent state. Let *d_k_* denote the drift state at time step *k*. Its temporal evolution is described by Equation (8).

(8) $$d_{k}=F_{k}d_{k-1}+q_{k}$$

where *F_k_* is the state transition coefficient governing the evolution of drift, and *q_k_* is the process noise associated with drift evolution.

Because drift generally changes slowly over time, Equation (8) can characterize the cumulative and persistent nature of long-term baseline deviation. Under a random-walk assumption, *F_k_* may be taken as 1.

#### 2.5.3. Construction of Drift Observation

Since the true drift cannot be directly measured, an observable quantity must first be constructed. In the present study, the drift observation is defined as the deviation between the cleaned measured temperature and the drift-free reference predicted by the IPSO-BP model, namely

(9) $$z_{k}=T_{k}^{\left(c\right)}-{\hat{T}}_{k}^{\left(BP\right)}$$

where *z_k_* represents the observable deviation attributed to sensor drift.

Equation (9) establishes the essential connection between the IPSO-BP prediction module and the recursive drift-estimation module in Figure 1. If the cleaned measurement ${\hat{T}}_{k}^{\left(c\right)}$ remains consistent with the predicted drift-free response ${\hat{T}}_{k}^{\left(\mathrm{BP}\right)}$, the constructed drift observation *z_k_* remains small. Conversely, when long-term drift exists, the deviation *z_k_* gradually accumulates and can then be tracked through recursive estimation.

#### 2.5.4. Recursive Drift Estimation and Corrected Temperature Output

Based on the constructed drift observation, the measurement equation for drift estimation is written as Equation (10).

(10) $$z_{k}=H_{k}d_{k}+r_{k}^{\left(d\right)}$$

where *H_k_* denotes the observation coefficient linking the latent drift state to the constructed observation, and *r_k_*^(d)^ denotes the observation noise in the drift-estimation stage. In the simplest case, *H_k_* may be taken as 1.

Using Equations (8) and (10), the IACKF recursively estimates the drift state. Let ${\hat{d}}_{k}$ denote the estimated drift at time step *k*. The final drift-corrected temperature is then obtained as

(11) $$T_{k}^{\left(\mathrm{corr}\right)}=T_{k}^{\left(c\right)}-{\hat{d}}_{k}$$

where *T_k_*^(corr)^ denotes the final corrected temperature after blind drift calibration.

Therefore, the IPSO-BP and IACKF modules play different yet complementary roles. The IPSO-BP model provides a reliable drift-free reference by learning the spatiotemporal correlation among adjacent monitoring points, while the IACKF converts the deviation between the cleaned measurement and the predicted reference into a recursively estimated drift state. Their integration enables long-term drift correction without requiring any external reference sensor.

### 2.6. Evaluation Metrics

To evaluate the performance of the proposed framework, different metrics are adopted for different anomaly-mitigation tasks. For noise reduction, the root mean square error (RMSE) and mean absolute percentage error (MAPE) are used to quantify the deviation between the estimated values and the reference values:

(12) $$\mathrm{RMSE}=\sqrt{\frac{1}{n}\sum_{i=1}^{n}{\left(y_{i}-{\hat{y}}_{i}\right)}^{2}}$$

(13) $$\mathrm{MAPE}=\frac{100\%}{n}\sum \left|\frac{{\hat{y}}_{i}-y_{i}}{y_{i}}\right|$$

where *y_i_* and ${\hat{y}}_{i}$ denote the reference value and the estimated value, respectively, and *n* is the number of samples.

The coefficient of determination *R*^2^ was used to evaluate the agreement between the processed results and the reference sequence:

(14) $$R^{2}=1-\frac{\sum_{i=1}^{n}(y_{i}-{\hat{y}}_{i}{)}^{2}}{\sum_{i=1}^{n}(y_{i}-\bar{y}{)}^{2}}$$

where $\bar{y}$ is the mean of the reference values.

The mean absolute error (MAE) and maximum absolute error (MaxAE) were additionally used to evaluate the average and worst-case deviations, respectively.

## 3. Experimental Setup and Data Description

### 3.1. Experimental Design

To comprehensively evaluate the proposed framework, three groups of experiments were designed with progressively increasing realism. Experiment I used simulated data generated from a nonlinear discrete dynamic system to verify the denoising capability of the IACKF under controlled time-varying Gaussian noise. Experiment II used measurements collected from a thermal resistance temperature sensor placed in a constant-temperature chamber to further assess the denoising performance of the IACKF under a controlled physical environment. Experiment III used field-monitored temperature data collected from a long-span cable-stayed bridge to evaluate the proposed multi-anomaly mitigation framework under practical SHM conditions. The field data were used to examine the denoising performance of the IACKF, the outlier-suppression performance of the OR-IACKF, and the drift-calibration performance of the IPSO-BP-IACKF. In addition, a semi-synthetic jointly contaminated dataset was constructed from the operation-stage measurements in Experiment III to evaluate the comprehensive performance of the complete framework under coexisting noise, outliers, and drift.

This experimental design follows a progressive verification strategy. The simulated data isolate the adaptive denoising mechanism under controlled noise conditions, the constant-temperature chamber data provide a controlled physical measurement scenario, and the field-monitored bridge data verify the applicability of the individual modules under realistic monitoring conditions. The semi-synthetic jointly contaminated dataset further enables a controlled assessment of the complete framework when multiple anomaly types coexist. Therefore, the three experiments, together with the extended jointly contaminated dataset, provide a progressive validation chain from numerical simulation and controlled measurements to field monitoring and comprehensive multi-anomaly mitigation.

#### 3.1.1. Experiment I: Simulated Nonlinear Dynamic System Data

A first-order nonlinear discrete dynamic system was adopted to generate simulated data for evaluating the denoising capability of the IACKF under a controlled condition. The system state equation and observation equation are given by

(15) $$x_{k}=0.5x_{k-1}+\frac{0.2x_{k-1}}{1+x_{k-1}^{2}}+8\cos\left[1.2\left(k-1\right)\right]+w_{k-1}$$

(16) $$z_{k}=\frac{x_{k}^{2}}{20}+v_{k}$$

where *x_k_* denotes the true system state at time step *k*, *z_k_* denotes the corresponding observation.

The number of simulation steps was set to 200. The theoretical initial state was set to 2, the initial state estimate was set to 2.1, and the initial error covariance was set to 0.01. For the standard CKF, the noise statistics were predefined, whereas the IACKF updated the noise covariance adaptively during the recursive process. Since the true state was known in this experiment, the denoising performance of the two methods could be quantitatively evaluated using the estimation error, RMSE, and MAPE.

#### 3.1.2. Experiment II: Constant Temperature Chamber Measured Data

In Experiment II, a thermal resistance temperature sensor was placed in a constant-temperature chamber maintained at 20 °C. The temperature was recorded for 200 sampling steps with a time interval of 1 s. The measured data were used to further evaluate the denoising performance of the IACKF under a controlled physical environment. The initial state estimate was set to 20.1. The standard CKF, the IACKF, and several classical denoising algorithms were applied for comparison.

To examine the influence of different initial settings on filtering performance, several combinations of the initial state estimate ${\hat{x}}_{0}$ and the initial error covariance *P*_0_ were considered in the chamber experiment. The corresponding parameter combinations are listed in Table 1. In particular, the combinations were designed to compare the effects of different initial estimated temperatures and different initial covariance levels on the convergence and stability of the recursive estimation process. The parameter combinations listed in Table 1 were subsequently used in the field-data robustness analysis presented in Section 4.1.4.

Because the chamber temperature remained essentially constant during the test, this experiment provided a suitable benchmark for evaluating the fluctuation-suppression capability of the different filtering methods. Compared with the simulated-data experiment, this test included actual sensor behavior and practical measurement uncertainty, and therefore offered a more realistic validation of the adaptive denoising strategy.

#### 3.1.3. Experiment III: Field-Measured Data from a Long-Span Cable-Stayed Bridge

**Figure 2:**
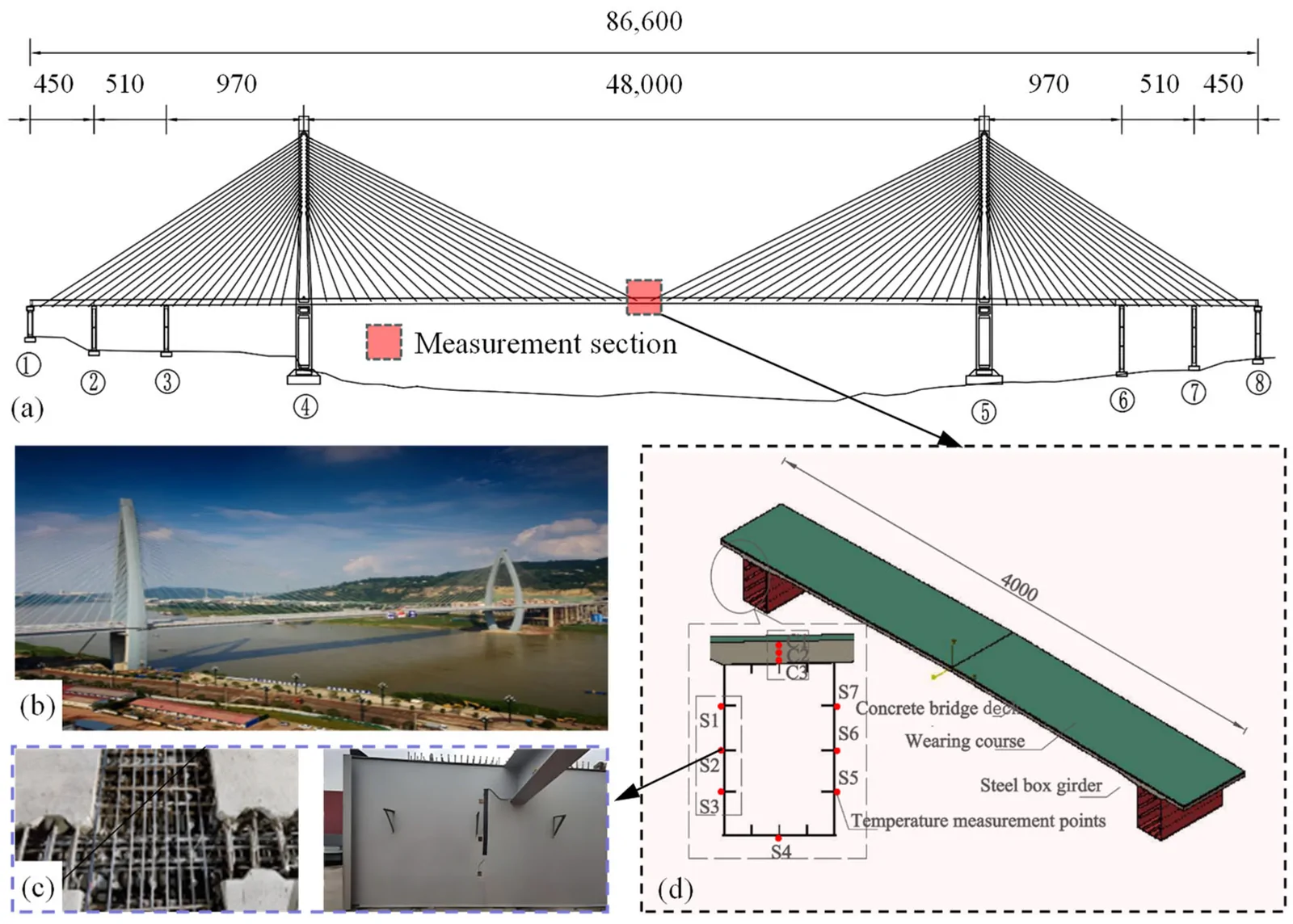
Bridge overview and temperature measurement arrangement (cm): (**a**) Location of the measurement section; (**b**) photograph of the investigated bridge; (**c**) field installation of temperature sensors; (**d**) cross-sectional configuration of the steel box girder and layout of temperature measurement points.

Experiment III was conducted using field-monitored temperature data collected from a long-span hybrid composite girder cable-stayed bridge with a main span of 480 m. The temperature sensors were installed in the 10# closure segment to monitor the temperature variation in different structural components, including the concrete deck, steel box girder, and pavement layer. According to the monitoring system configuration, the sensor model was HYT-ADS11XX, with a measurement range of −40 °C to +125 °C and an accuracy of ±0.5 °C. The geometric dimensions of the closure segment and the layout of the monitoring points are shown in Figure 2 of the manuscript. The sensors were labeled C1~C3 for the concrete deck and S1~S7 for the steel box girder and pavement layer.

This field experiment was designed to evaluate the applicability of the proposed framework under realistic SHM conditions. Unlike the simulated data and chamber data, the bridge monitoring data reflect the combined influence of environmental loading, sensor uncertainty, construction-stage effects, and long-term operational disturbance. Therefore, they provide a practical basis for validating the proposed multi-anomaly mitigation framework in real bridge temperature monitoring.

Two groups of field-monitored data were selected from different bridge stages. The first group corresponded to the hydration heat period during construction, in which the data were sampled every 7.5 min over 24 h, resulting in 192 samples. The monitoring points involved in this stage were C1, C3, and S4. The second group corresponded to the bridge operation period under high-temperature environmental conditions, in which the data were sampled every 15 min over 240 h, resulting in 960 samples. The monitoring points involved in this stage were C2, S3, S4, and S6. The measurement parameter settings for the two field-monitoring periods are summarized in Table 2. In addition, synchronized operation-stage measurements from C1 and C3 were used as auxiliary reference inputs for constructing the proxy clean reference in the jointly contaminated dataset.

The hydration heat dataset was used to characterize temperature evolution during the construction stage, while the operation-stage dataset was used to represent long-term temperature variation under service conditions. Since field-monitored bridge temperature data may simultaneously contain random noise, outliers, and drift, this experiment was used to validate the full collaborative framework, including adaptive denoising, robust outlier suppression, and blind drift calibration.

### 3.2. Construction of the Jointly Contaminated Dataset

To evaluate the proposed framework under multiple coexisting anomalies, a semi-synthetic dataset was constructed using operation-stage bridge temperature measurements. The C2 sequence was selected as the target signal, while C1 and C3 were used as reference measurements. A proxy clean reference was generated by combining the ridge-regression prediction based on C1 and C3 with the robust C2 trend obtained using Hampel filtering and Savitzky–Golay (SG) smoothing. Gaussian noise, isolated outliers, patch-type outliers, and two artificial drift events characterized by rapid onset, short-term persistence, and gradual recovery were then superimposed on the proxy reference.

## 4. Experimental Results and Analysis

### 4.1. Noise Reduction Performance

#### 4.1.1. Comparison with the Standard CKF Under Time-Varying Noise

To evaluate the adaptive denoising capability of the IACKF, simulated data generated from a first-order nonlinear discrete dynamic system were used. In contrast to the constant-noise condition, a time-varying noise scenario was constructed to represent the uncertainty commonly encountered in practical monitoring applications.

The process-noise covariance was defined as

(17) $$Q_{k}=\left\{\begin{array}{cc} 2.0, & 1\leq k\leq 70,\\ 5.0, & 71\leq k\leq 130,\\ 1.0, & 131\leq k\leq 200, \end{array}\right.$$

and the observation-noise covariance was defined as

(18) $$R_{k}=\left\{\begin{array}{cc} 1.0, & 1\leq k\leq 100,\\ 2.5, & 101\leq k\leq 200. \end{array}\right.$$

**Figure 3:**
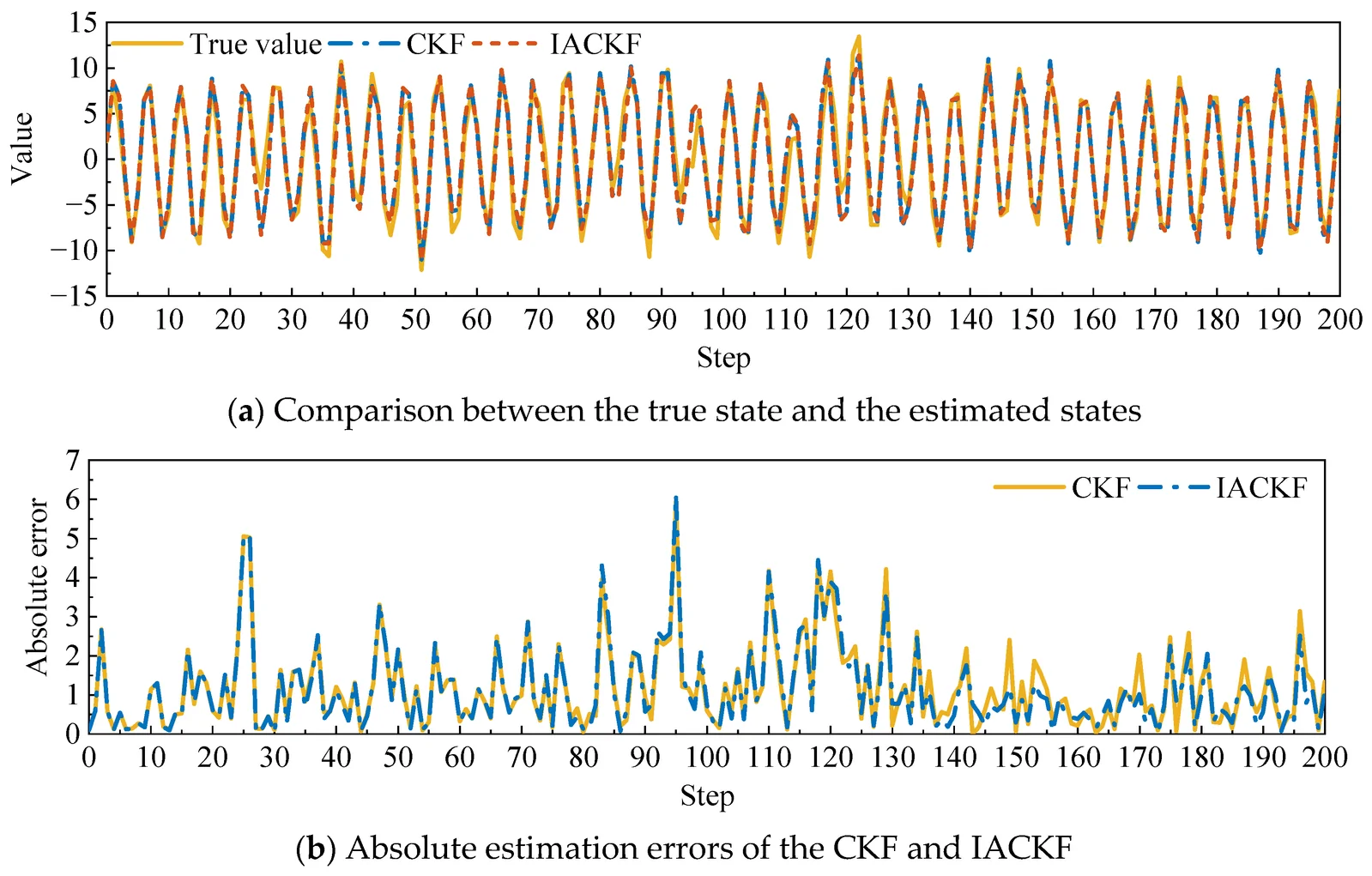
State-estimation results under time-varying noise.

For a fair comparison, the standard CKF and the IACKF used the same simulated states, observations, initial state estimate, initial error covariance, and initial noise-covariance values. The standard CKF retained fixed covariance values throughout the recursive process, whereas the IACKF updated the process- and observation-noise covariances online using the innovation and residual information. The state-estimation trajectories and absolute errors obtained in a representative simulation are shown in Figure 3.

**Figure 4:**
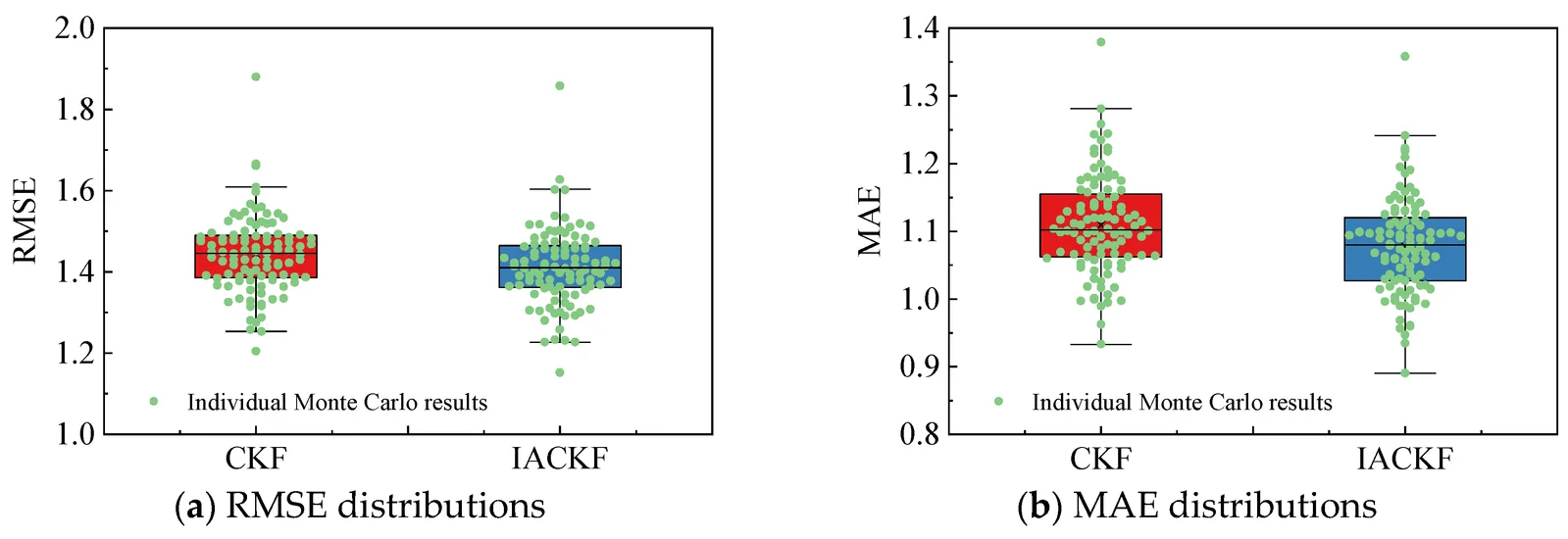
Statistical comparison based on 100 Monte Carlo simulations.

Both algorithms were able to track the nonlinear state evolution, while the IACKF exhibited smaller estimation deviations after the noise statistics changed. To reduce the influence of an individual random-noise realization, 100 independent Monte Carlo simulations were further conducted. The RMSE and MAE distributions are presented in Figure 4. The IACKF achieved lower RMSE than the standard CKF in 89% of the simulations and lower MAE in 94% of the simulations. These results indicate that the improvement was not caused by a favorable random-noise realization, but was consistently observed under time-varying noise conditions.

#### 4.1.2. Verification Using Constant-Temperature Chamber Data

To further validate the practical denoising performance of the IACKF, measured data collected from a constant-temperature chamber were used for comparison with several conventional and adaptive filtering methods. A thermal resistance temperature sensor was placed in a chamber maintained at 20.0 °C, and 200 measurements were continuously recorded at a sampling interval of 1 s. Based on the fluctuation characteristics of the measured sequence and the controlled chamber condition, the initial observation-noise covariance and process-noise covariance were set to ${\hat{R}}_{0}$ = 0.05 and ${\hat{Q}}_{0}$ = 0.01, respectively. These values correspond to standard deviations of approximately 0.224 °C and 0.100 °C. The same initial covariance settings were adopted for the CKF and IACKF to ensure a consistent comparison.

For comparison, the moving average (MA), (SG), CKF, and Sage–Husa adaptive cubature Kalman filter (SH-ACKF) were also applied to the same chamber dataset. The MA and SG methods represent conventional smoothing approaches, the CKF uses fixed noise covariances, and the SH-ACKF represents an adaptive covariance-estimation method. All methods were evaluated using the same observations and reference temperature.

**Figure 5:**
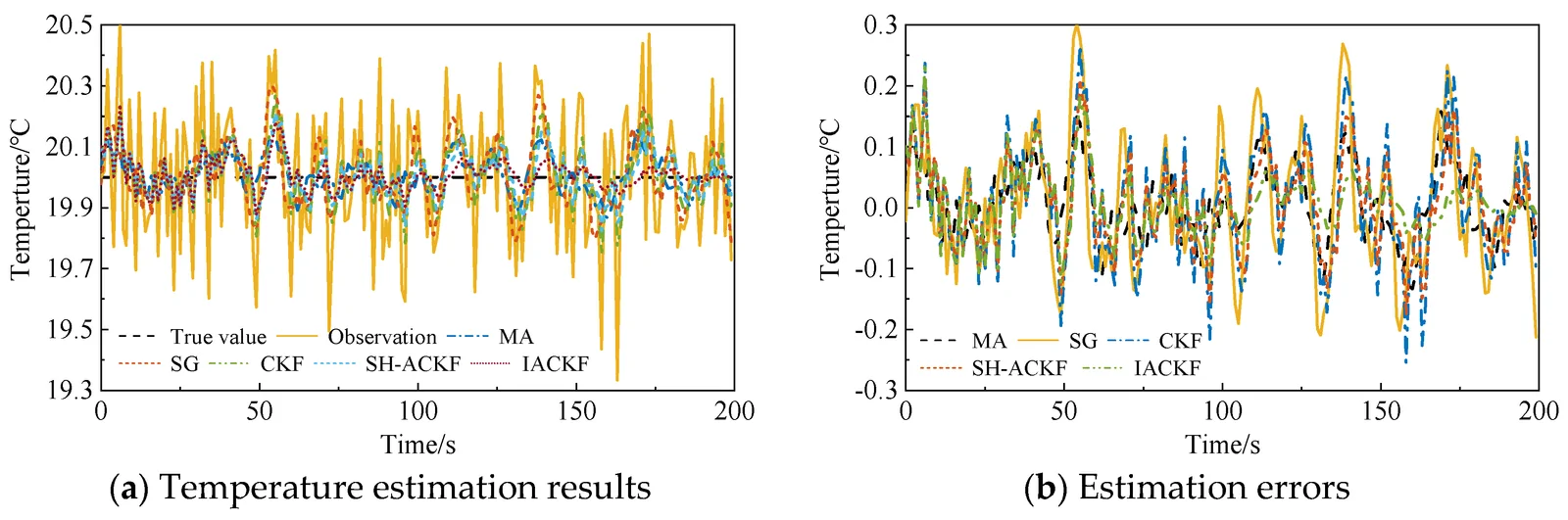
Comparison of temperature estimates and estimation errors obtained by different denoising methods in Experiment II.

**Figure 6:**
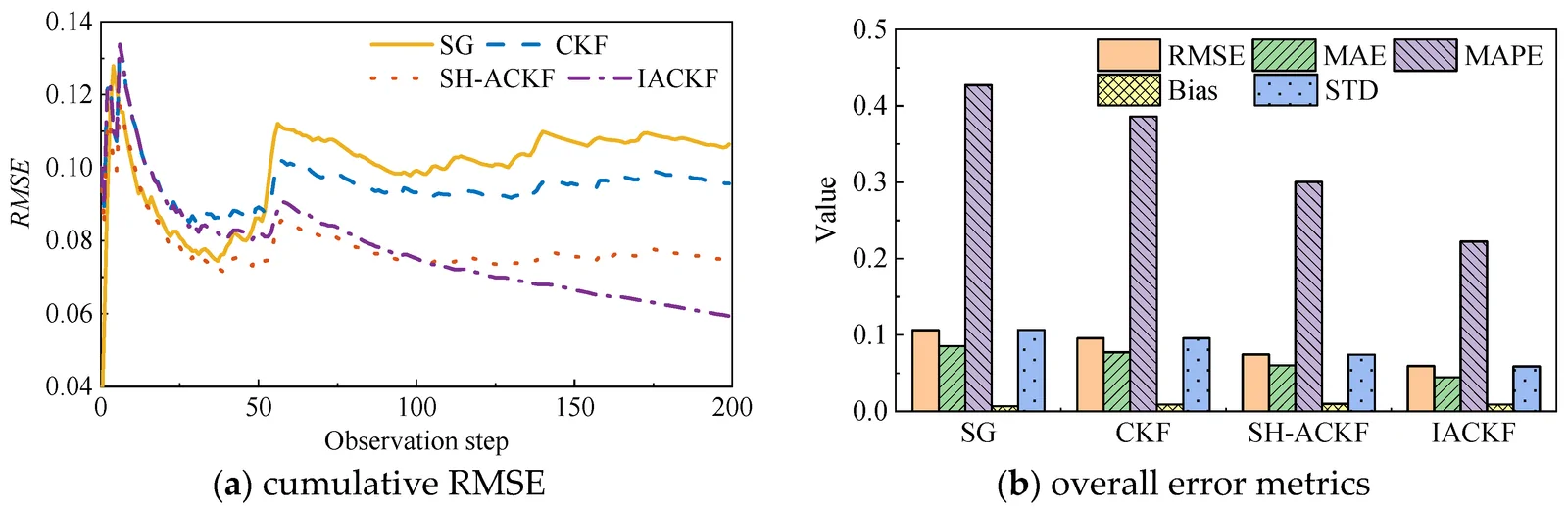
Quantitative comparison of denoising performance for different methods in Experiment II.

No artificial drift was introduced because this experiment was designed specifically to evaluate denoising performance under a controlled constant-temperature condition. The corresponding results are shown in Figure 5 and Figure 6.

As shown in Figure 5, all methods reduced the short-term fluctuations in the measured temperature sequence to different degrees. The MA and SG methods provided basic smoothing effects, while the CKF and SH-ACKF further improved the continuity of the estimated temperature curves. Compared with these methods, the IACKF produced a smoother estimate with smaller local fluctuations while maintaining the overall temperature level near 20.0 °C. The corresponding error curves also show that the IACKF generally yielded a narrower error range. Figure 6 further indicates that the cumulative RMSE of the IACKF gradually decreased and remained relatively stable as the number of observations increased. The overall error indicators also suggest that the IACKF achieved comparatively lower RMSE, MAE, MAPE, and standard deviation (STD), while maintaining a small bias. These results indicate that the adaptive covariance-update mechanism improves the suppression of measurement disturbances under the constant-temperature condition. Compared with the numerical simulation, the chamber experiment provides a more realistic validation because it includes actual sensor behavior and measurement uncertainty. Therefore, the results further support the use of the IACKF as the first-stage denoising method in the proposed framework.

#### 4.1.3. Field Validation Under Hydration and Operation Conditions

**Figure 7:**
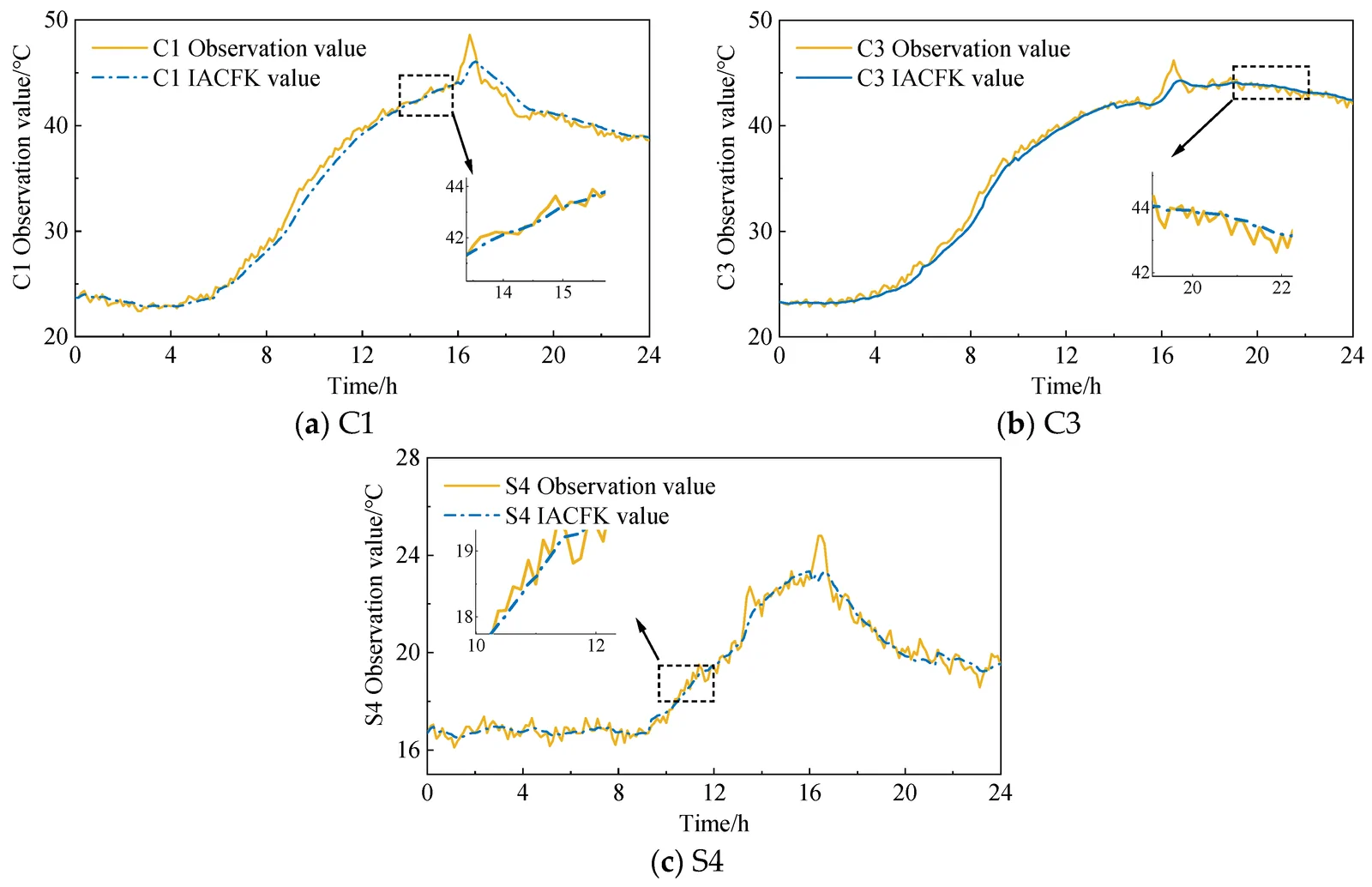
IACKF denoising results during the hydration heat period.

**Figure 8:**
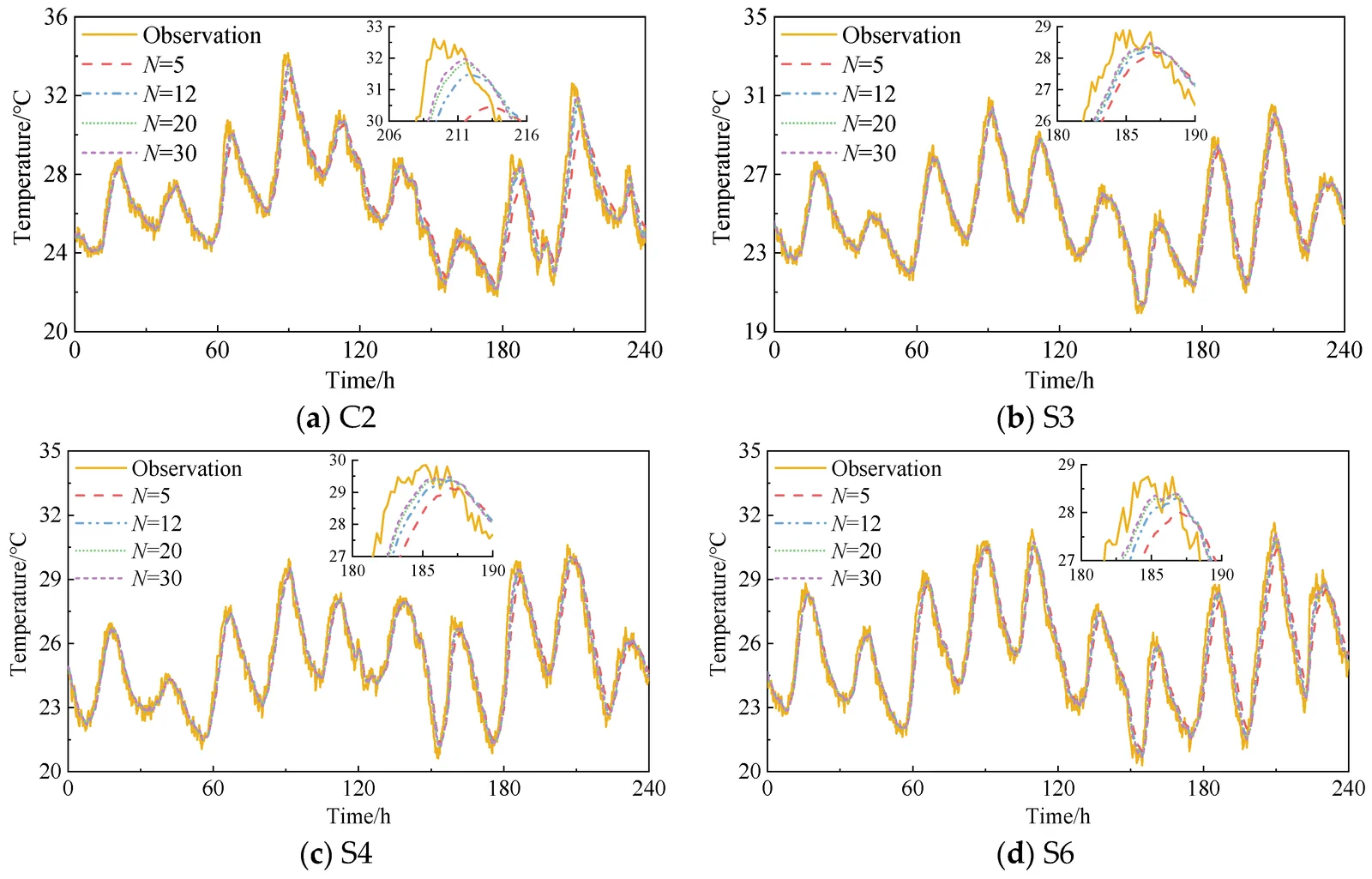
Influence of the sliding-window length on the IACKF denoising results during the operation period.

The field performance of the IACKF was evaluated using bridge temperature monitoring data collected during the hydration heat and operation periods. The hydration heat dataset included monitoring points C1, C3, and S4, whereas the operation-stage dataset included monitoring points C2, S3, S4, and S6. Comparisons between the raw observations and the IACKF-filtered results are presented in Figure 7 and Figure 8.

As shown in Figure 7, during the hydration heat period, the IACKF effectively preserved the overall temperature evolution trend while suppressing local fluctuations in the measured data. Consistent with the observations at monitoring points C1 and C3, the filtered sequences remained highly consistent with the observed temperature rise caused by hydration heat, while the local abnormal oscillations were weakened after filtering. A similar effect can be observed at point S4, where the IACKF smoothed the short-term fluctuations without altering the main thermal development pattern. These results indicate that the IACKF can reduce random disturbance while preserving the physically meaningful temperature evolution during the construction stage.

As shown in Figure 8, the IACKF effectively suppressed the local fluctuations in the operation-stage temperature data while preserving the overall cyclic variation patterns at C2, S3, S4, and S6. The filtered results obtained with different sliding-window lengths were generally consistent with the measured temperature trends, but noticeable differences appeared near rapidly varying temperature segments. A smaller window length, such as *N* = 5, provided a faster response but produced relatively larger local fluctuations. As *N* increased, the filtered curves became smoother and more stable. However, an excessively large window, such as *N* = 30, resulted in a more evident response delay near some temperature peaks and valleys. Overall, *N* = 20 provided a suitable balance between filtering stability and trend-tracking capability.

**Figure 9:**
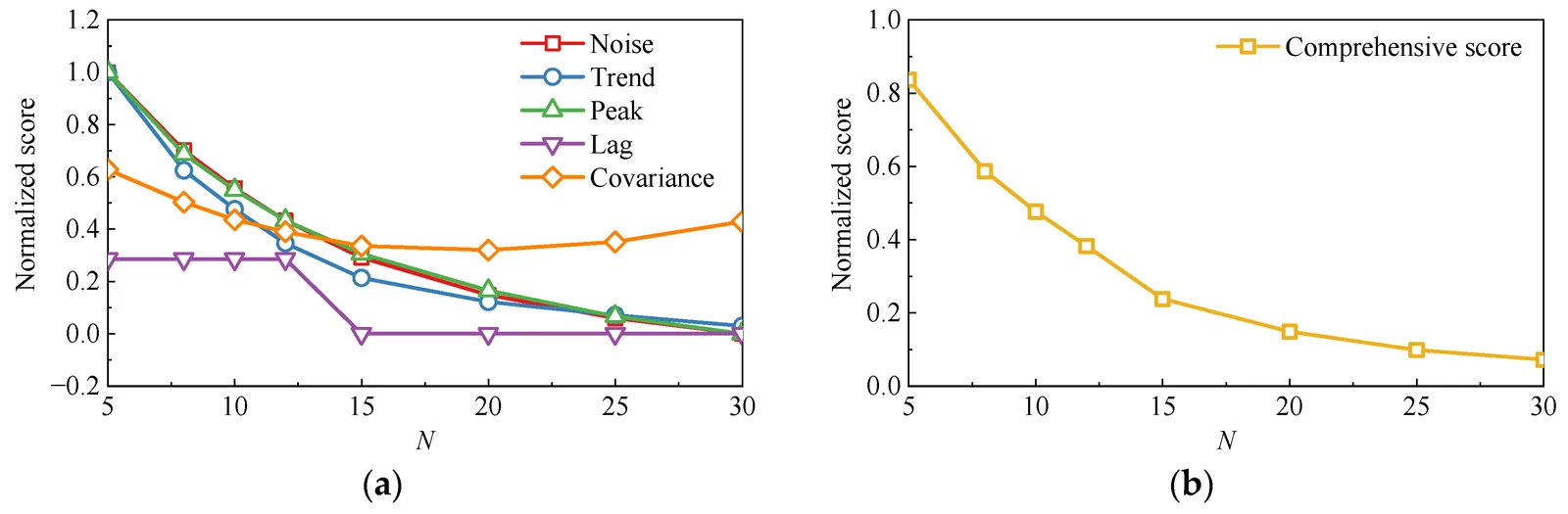
Sensitivity analysis and selection of the sliding-window length *N.* (**a**) normalized performance indicators under different window lengths; (**b**) multi-sensor comprehensive score and elbow-point analysis.

To quantitatively determine the sliding-window length, values of *N* = 5, 8, 10, 12, 15, 20, 25, and 30 were evaluated using the field data from all monitoring points. Lower normalized scores indicate better performance. As shown in Figure 9a, the normalized noise, trend, and peak-related scores generally decreased as *N* increased, indicating improved smoothing and trend preservation. The lag-related score remained nearly unchanged for *N* ≥ 15, whereas the covariance-related score reached its minimum at approximately *N* = 20 and increased thereafter, indicating that an excessively large window may reduce the adaptability of the covariance update. Although the comprehensive score in Figure 9b continued to decrease slightly beyond *N* = 20, the improvement became limited, while larger windows produced more evident response delays near rapidly varying temperature peaks and valleys. Therefore, *N* = 20 was selected as a suitable compromise among filtering stability, covariance adaptability, and trend-tracking responsiveness.

#### 4.1.4. Robustness to Initial State and Error Covariance Settings

**Figure 10:**
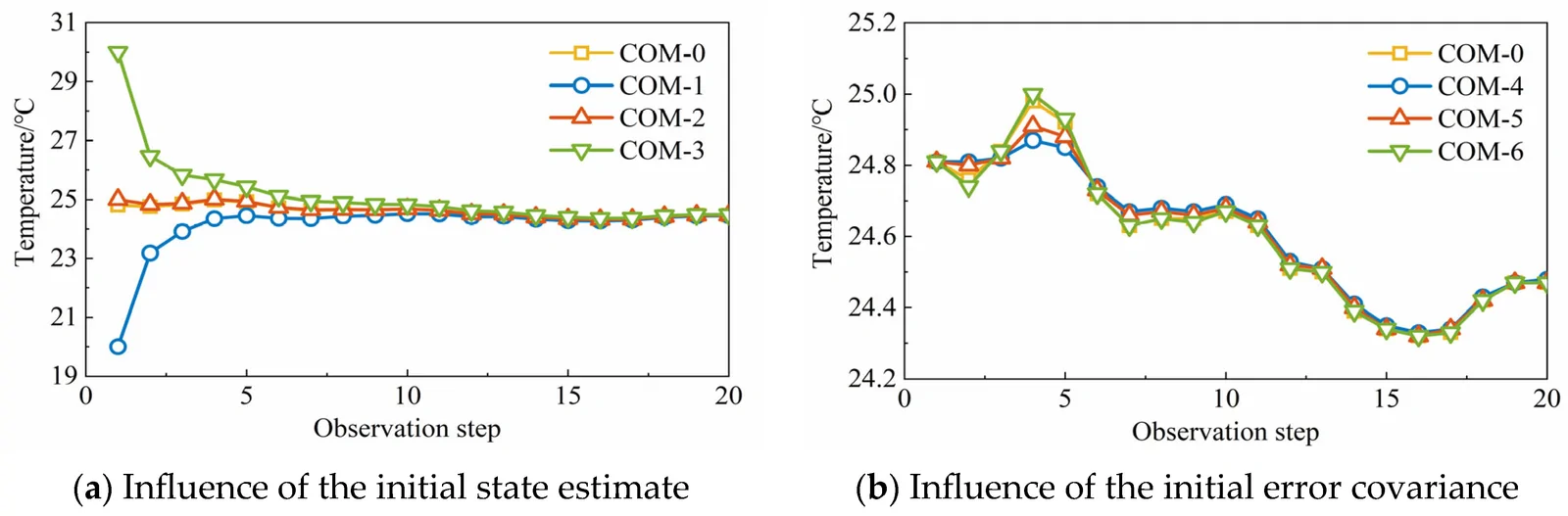
Robustness of the IACKF to different initial-state and initial-covariance settings for C2.

**Figure 11:**
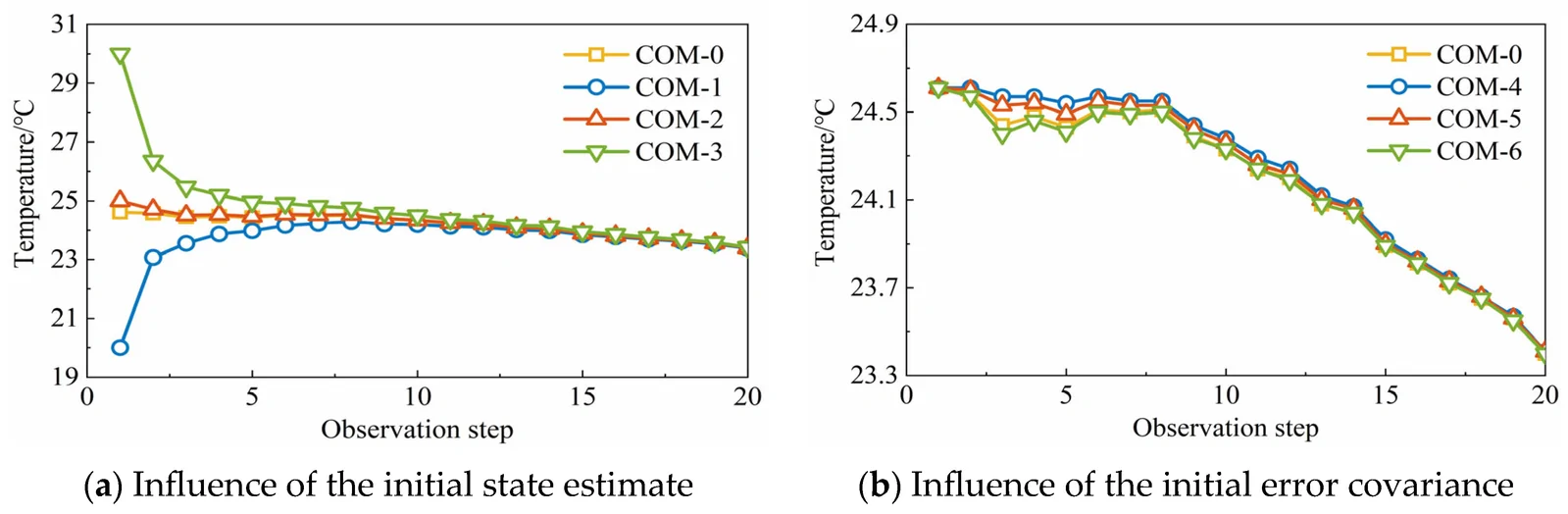
Robustness of the IACKF to different initial-state and initial-covariance settings for S3.

In the above denoising analysis, the initial state estimate ${\hat{x}}_{0}$ and the initial error covariance *P*_0_ were assigned fixed values. To further examine the robustness of the IACKF to different initial parameter settings, the operation-stage monitoring data at points C2 and S3 were selected as representative examples. Different combinations of the initial state estimate ${\hat{x}}_{0}$ and the initial error covariance *P*_0_ were considered, as summarized in Table 1, and the denoising results for the first 20 observation steps are shown in Figure 10 and Figure 11. The parameter combination COM-0 corresponds to the initial setting adopted in Figure 8.

As shown in Figure 10 and Figure 11, different initial settings mainly affect the denoising performance in the early stage of the observation sequence. With the increase in the observation step, the filtered results under different parameter combinations gradually converge and become highly consistent. This indicates that the IACKF has good robustness to the initial values of ${\hat{x}}_{0}$ and *P*_0_. In particular, the initial state estimate ${\hat{x}}_{0}$ has a more visible influence on the denoising results in the first several observation steps, whereas the influence of the initial error covariance *P*_0_ is relatively limited.

Therefore, in practical applications of the IACKF, more attention should be paid to the initialization of the early-stage state estimate. When necessary, a short warm-up sequence may be introduced at the beginning of the monitoring process to reduce the influence of the initial parameter settings on the denoising quality. Overall, the results in Figure 10 and Figure 11 confirm that the IACKF maintains stable denoising performance under different initial parameter settings, demonstrating good robustness for practical monitoring applications.

### 4.2. Outlier Suppression Performance

**Figure 12:**
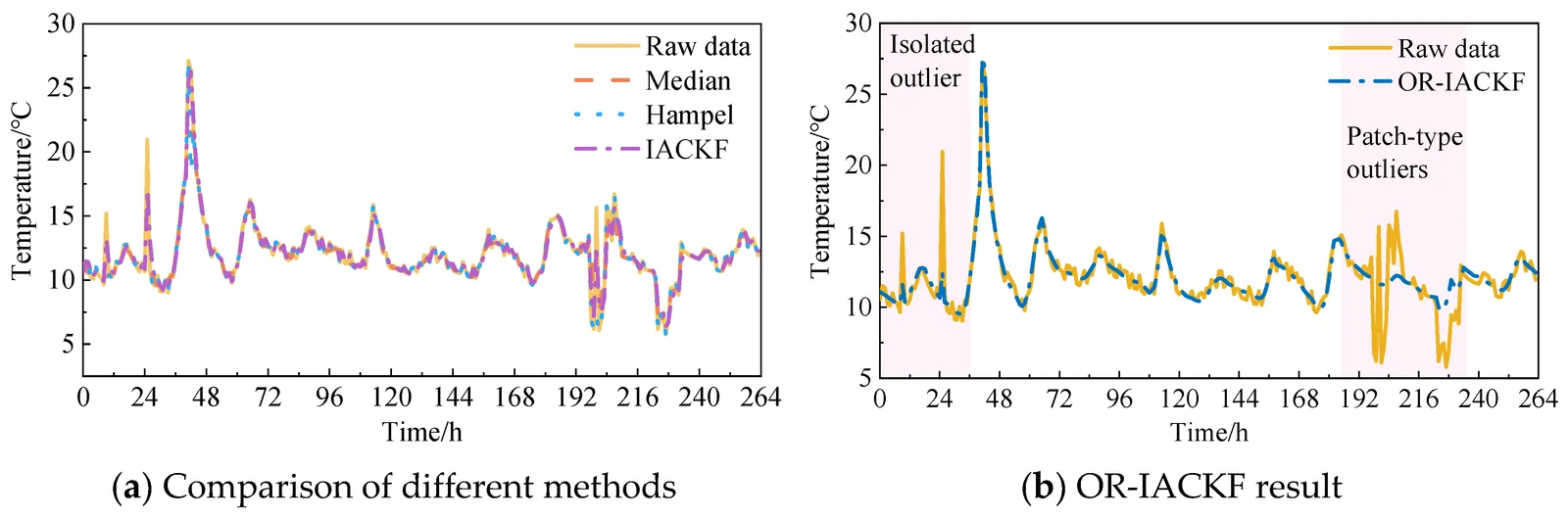
Outlier suppression results for the S1 temperature series.

**Figure 13:**
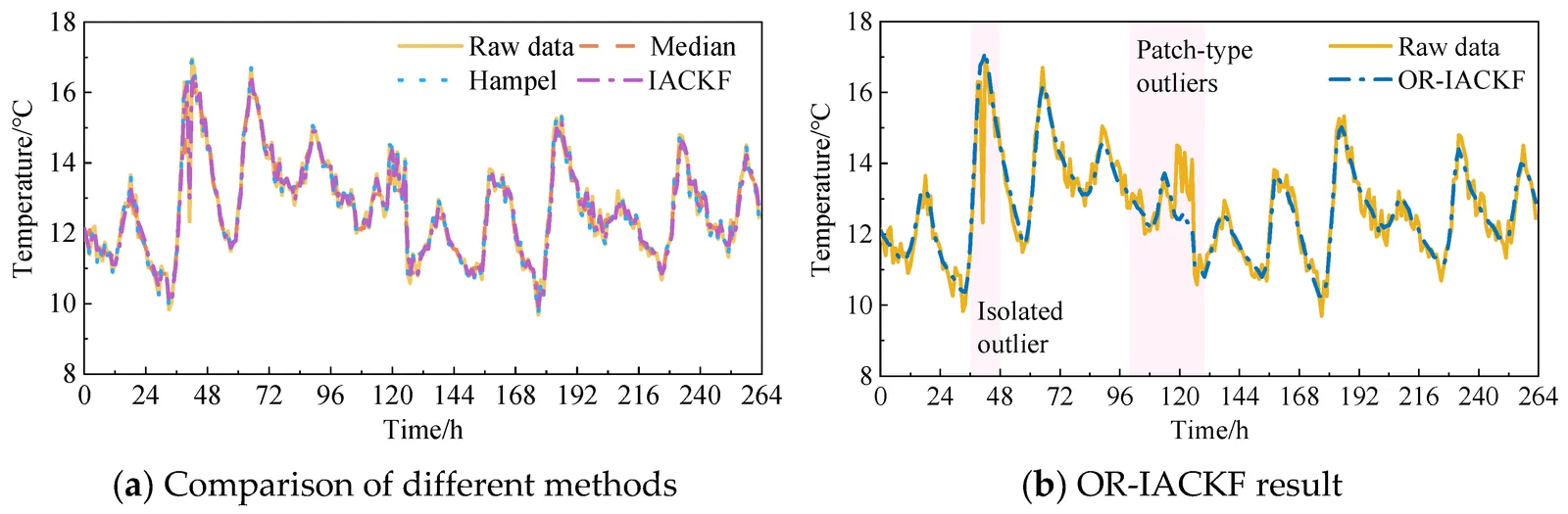
Outlier suppression results for the C1 temperature series.

To evaluate the outlier suppression capability of the OR-IACKF, the field temperature records collected at monitoring points S1 and C1 from 00:00 on 21 January 2021 to 23:00 on 31 January 2021 were selected. Both isolated and patch-type outliers were present in the selected sequences. The initial state estimate and error covariance were set as ${\hat{x}}_{0}=z_{0}$, $P_{0}=1$, respectively. For the contaminated Gaussian observation model, the prior probabilities were set to $p_{1}=0.85$, $p_{2}=0.15$, and the variance amplification factor was set to q=5. To provide a broader comparison, the Median filter, Hampel filter, and standard IACKF were applied to the same datasets. The Median filter suppresses impulsive disturbances using the local median, whereas the Hampel filter detects abnormal observations based on the local median and median absolute deviation. These methods were selected as representative conventional outlier-processing approaches, while the IACKF was used to evaluate the improvement introduced by the robust observation model. Figure 12 and Figure 13 present the outlier suppression results for S1 and C1, respectively.

In Figure 12a and Figure 13a, the Median and Hampel filters reduced part of the abrupt fluctuations but introduced different degrees of local smoothing. The standard IACKF preserved the overall temperature evolution more effectively; however, it remained sensitive to isolated and patch-type outliers, especially when consecutive abnormal observations occurred.

Figure 12b and Figure 13b show the corresponding OR-IACKF results, with the isolated- and patch-type outlier intervals highlighted. For S1, the OR-IACKF substantially weakened the influence of abnormal observations and maintained good continuity with the surrounding temperature trend. A similar improvement was observed for C1. Although the fluctuation pattern of C1 differed from that of S1, the OR-IACKF effectively suppressed both isolated and clustered abnormal deviations without visibly distorting the main temperature evolution.

Because the true outlier-free values of the field measurements were unavailable, the results were evaluated qualitatively rather than using error indicators against an assumed reference. The comparison shows that the contaminated Gaussian observation model effectively reduces the contribution of abnormal measurements without excessively smoothing valid temperature variations. The results also indicate coupling between background noise and outliers. Noise reduces the distinction between normal and abnormal innovations, while outliers may contaminate the adaptive covariance estimates. By reducing the contribution of high-likelihood outliers and restricting covariance adaptation within abnormal intervals, the OR-IACKF limits this mutual interference.

### 4.3. Blind Drift Calibration Performance

**Figure 14:**
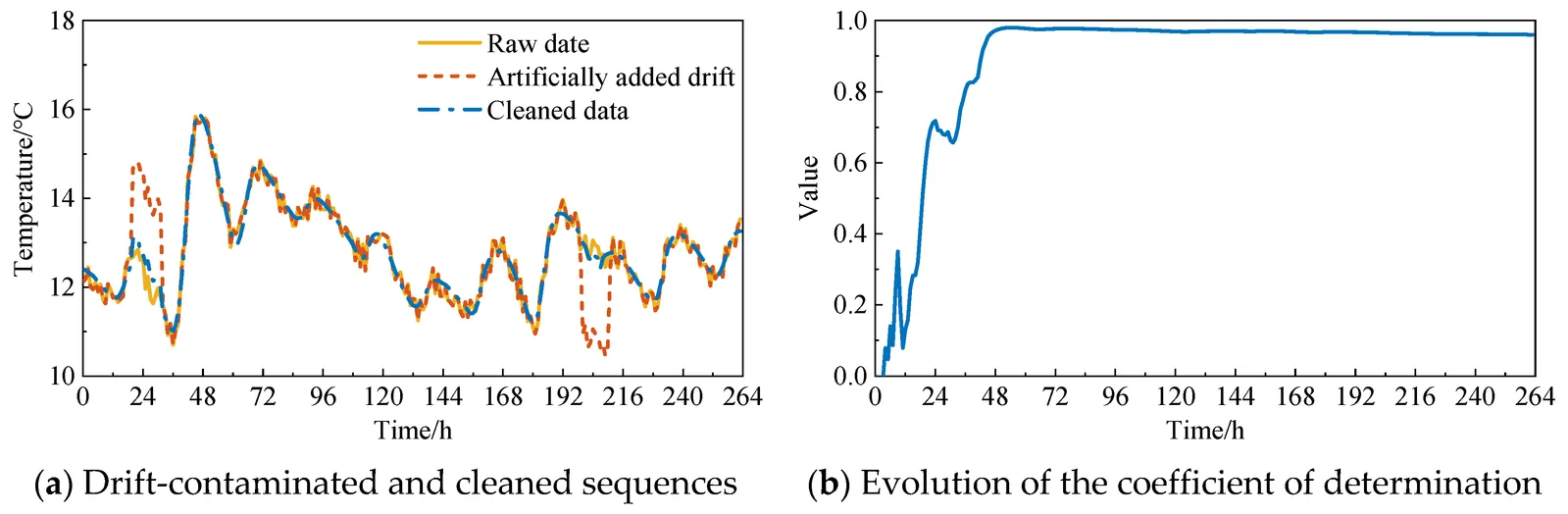
Drift calibration results of the IPSO-BP-IACKF framework.

To evaluate the drift-calibration performance of the IPSO-BP-IACKF framework, C2 was selected as the target monitoring point, while C1 and C3 were used as adjacent reference points. Short-term measurements collected during the initial drift-free period were used for model training, and long-term measurements collected from 00:00 on 21 January 2021 to 23:00 on 31 January 2021 were used for validation. The sampling interval was 15 min. Because the true sensor drift in field monitoring was unavailable, artificial drift was superimposed on the long-term C2 temperature sequence to enable quantitative evaluation of the drift-correction performance. The initial parameters of the IPSO-BP-IACKF were set as *n* = 2, *l* = 5, *m* = 1, *α* = 1, and *K* = 100, with two hidden layers. The coefficient of determination, R^2^, was adopted as the evaluation metric. The corrected temperature sequence and the corresponding evaluation results are presented in Figure 14.

As shown in Figure 14a, the artificially introduced drift results in noticeable deviations from the original temperature sequence in several segments, whereas the cleaned data produced by the IPSO-BP-IACKF framework remain closely aligned with the underlying temperature trend. The abnormal baseline deviation is effectively eliminated, and the corrected sequence accurately recovers the intrinsic temperature evolution. These results demonstrate that the proposed method can reliably identify and compensate for slowly varying drift components.

The effectiveness of the cleaning result can be explained by the two-level structure of the proposed framework. On the one hand, the IPSO-BP model provides a drift-free reference response by learning the spatiotemporal correlation between the target point and adjacent monitoring points. On the other hand, the IACKF converts the deviation between the drift-contaminated sequence and the predicted reference into a recursively estimated drift state, thereby enabling gradual correction of the accumulated baseline error. As a result, the proposed method is well suited to the slow and persistent characteristics of sensor drift in long-term bridge temperature monitoring.

Figure 14b shows the evolution of the coefficient of determination R^2^ with the observation number. It can be seen that the evaluation index increased rapidly during the early stage of the observation sequence and then gradually stabilized at a high level close to 1.0. The coefficient of determination after drift calibration reached approximately 0.96, indicating strong agreement between the corrected sequence and the original drift-free temperature series.

This variation pattern suggests that the proposed framework quickly established a reliable calibration effect after the initial observation stage and then maintained stable cleaning performance over the long-term monitoring sequence. The rapid increase in the early stage reflects the progressive convergence of the recursive calibration process, while the subsequent stabilization indicates that the estimated drift state remained consistent with the actual drift-contaminated behavior. Therefore, the results in Figure 14b further confirm the effectiveness and stability of the IPSO-BP-IACKF framework for long-term drift correction.

Taken together, the results in Figure 14 demonstrate that the IPSO-BP-IACKF framework can effectively correct long-term drift in bridge temperature monitoring data. The method not only restores the target temperature sequence by removing the artificially introduced drift, but also achieves a stable and high evaluation performance over the observation process. Compared with ordinary denoising or outlier suppression alone, this strategy directly addresses the cumulative and persistent nature of drift and therefore extends the proposed framework from local anomaly mitigation to long-term baseline correction. These results demonstrate the potential applicability of the proposed method to long-term bridge temperature monitoring.

### 4.4. Joint Multi-Anomaly Mitigation Performance

To evaluate the comprehensive cleaning capability of the proposed framework, a semi-synthetic jointly contaminated dataset was constructed from the operation-stage field measurements. The C2 temperature series was selected as the target signal, while C1 and C3 were used as reference measurements. A proxy clean reference was constructed by combining the ridge-regression prediction based on C1 and C3 with the robust trend extracted from C2, using weights of 0.1 and 0.9, respectively. Two artificial drift events characterized by rapid onset, short-term persistence, and gradual recovery were introduced together with Gaussian noise and isolated and patch-type outliers.

**Figure 15:**
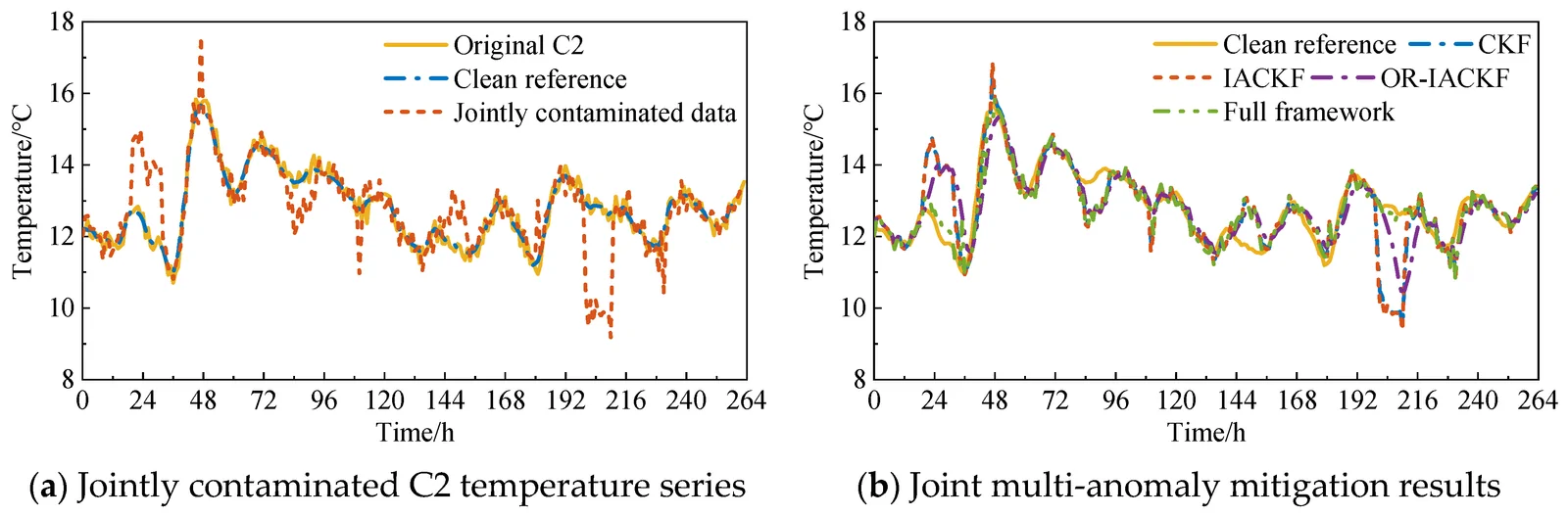
Construction and mitigation results of the jointly contaminated C2 temperature series.

The contaminated data were sequentially processed by the IACKF, OR-IACKF, and IPSO-BP-IACKF modules. The IACKF first reduced background noise, after which the OR-IACKF selectively suppressed abnormal observations. Finally, the IPSO-BP-IACKF drift-correction results were applied within the predefined drift intervals. This staged strategy reduces the influence of random noise on outlier suppression and limits the adverse effect of abnormal observations on drift calibration. As shown in Figure 15, the IACKF reduced high-frequency fluctuations, while the OR-IACKF further mitigated isolated and patch-type outliers. Nevertheless, noticeable baseline deviations remained within the drift-contaminated intervals. After drift correction, the final result was substantially closer to the proxy clean reference.

As summarized in Table 3, the proposed full framework achieved the best overall performance on the jointly contaminated dataset. The progressive comparison among CKF, IACKF, OR-IACKF, and the full framework illustrates the contribution of each processing module. The CKF reduced the RMSE from 0.855 °C for the contaminated data to 0.789 °C. The IACKF further reduced the MAE from 0.485 °C to 0.446 °C, although its RMSE was slightly higher than that of CKF, indicating that adaptive covariance updating alone was insufficient to address the coupled effects of noise, outliers, and drift. After introducing the dual-Gaussian contaminated observation model, the OR-IACKF reduced the RMSE to 0.675 °C and the MaxAE to 2.357 °C, demonstrating improved robustness against large abnormal deviations.

With the further incorporation of the drift-correction module, the proposed full framework increased R^2^ from 0.488 for OR-IACKF to 0.823 and reduced the RMSE, MAE, and MaxAE to 0.397, 0.266, and 1.491 °C, respectively. Compared with the raw jointly contaminated data, these values correspond to reductions of approximately 53.6%, 45.2%, and 57.4%. The results confirm that adaptive denoising, robust outlier suppression, and drift correction provide complementary improvements.

The staged processing strategy also helps reduce mutual interference among different anomaly types. Random noise increases innovation fluctuations and weakens the distinction between normal observations and outliers, while large outliers may contaminate the online covariance estimates. Slowly varying drift changes the residual baseline and may be misidentified as persistent local anomalies. By successively reducing background noise, suppressing abrupt outliers, and correcting the remaining drift component, the proposed framework limits the propagation of one anomaly type into the subsequent stages.

It should be noted that the present validation used a semi-synthetic dataset with predefined artificial outlier and drift intervals. Therefore, further verification using long-term field data and fully automatic anomaly identification is still required. Nevertheless, the recursive implementation of IACKF and OR-IACKF requires only limited historical data, and the IPSO-BP optimization can be completed offline. The trained model therefore has potential for near-real-time bridge temperature monitoring, although its performance on low-power edge devices still requires additional evaluation.

## 5. Conclusions

This study proposed a collaborative IACKF-based framework for multi-anomaly mitigation in bridge temperature monitoring data. The framework was developed to address three representative anomaly types in long-term monitoring sequences, namely random noise, outliers, and sensor drift. Based on simulated data, controlled measurements, and field-monitored bridge temperature data, the effectiveness of the proposed framework was systematically verified. The main conclusions are summarized as follows.

(1) The IACKF demonstrated effective adaptive denoising performance under the simulated time-varying-noise condition and the constant-temperature chamber experiment. By updating the process- and observation-noise covariance matrices online using innovation and residual information, the IACKF outperformed the standard CKF in both experiments and also showed good applicability to field-monitored bridge temperature data. In addition, the robustness analysis under different initial parameter settings indicated that the IACKF maintained stable denoising performance after the initial observation stage, demonstrating its robustness for practical monitoring applications.
(2) The OR-IACKF effectively suppressed isolated and patch-type outliers in bridge temperature monitoring data. By introducing a dual-Gaussian contaminated observation model and posterior weighting into the recursive update process, the OR-IACKF exhibited stronger robustness to abnormal observations than the IACKF for the C1 and S1 monitoring datasets containing artificial outliers. The results indicate that the robust observation mechanism can reduce the influence of contaminated measurements while preserving the underlying temperature variation trend.
(3) The IPSO-BP-IACKF framework can effectively correct long-term sensor drift. By using adjacent monitoring points to construct a drift-free reference response and then recursively estimating the drift state through the IACKF, the method successfully reduced the artificially introduced drift in the target monitoring sequence. The drift-cleaning evaluation metric increased rapidly and then stabilized at a high level, and the coefficient of determination after drift cleaning reached approximately 0.96, indicating strong calibration effectiveness and stability.
(4) The collaborative framework provides a progressive solution for multi-anomaly mitigation in bridge temperature monitoring data. The IACKF addresses random noise, the robust IACKF further handles non-Gaussian outliers, and the IPSO-BP-IACKF extends the framework to long-term drift correction. This staged design is well suited to the heterogeneous characteristics of practical monitoring anomalies and provides a feasible strategy for improving the reliability of long-term bridge temperature monitoring data.
(5) Although the framework demonstrates satisfactory performance in processing noise, isolated outliers, patch-type outliers, and drift in bridge temperature monitoring data, several engineering limitations remain. The identification and classification of some abnormal regions still rely on prior knowledge, and the algorithm parameters may need to be adjusted for different sensors and monitoring environments. Moreover, the current validation is limited to a relatively small number of monitoring cases. Future studies will focus on developing automatic anomaly-region and anomaly-type identification methods, incorporating spatial correlations among multiple sensors, and conducting long-term validation on different bridge types and environmental conditions to further improve the generalization and engineering applicability of the framework.

## Figures and Tables

**Table 1:** Initial estimate and error covariance combinations.

Combination No.	Initial Parameters	
	Initial State Estimate ${\hat{x}}_{0}$	Initial Error Covariance $P_{0}$
COM-0	$z_{0}$	1
COM-1	20	1
COM-2	25	1
COM-3	30	1
COM-4	$z_{0}$	0.01
COM-5	$z_{0}$	0.1
COM-6	$z_{0}$	10

**Table 2:** Measurement parameter settings.

Period	Sampling Interval	Duration	Sensor ID
Hydration heat period	7.5 min	24 h	C1, C3, S4
Operation period	15.0 min	240 h	C2, S3, S4, S6

**Table 3:** Quantitative comparison of different methods on the jointly contaminated dataset.

Method	R^2^	RMSE/°C	MAE/°C	MaxAE/°C
Jointly contaminated data	0.177	0.855	0.485	3.499
CKF	0.299	0.789	0.446	3.172
IACKF	0.277	0.802	0.446	3.308
OR-IACKF	0.488	0.675	0.439	2.357
MA filter	0.381	0.742	0.392	2.994
SG filter	0.329	0.772	0.407	3.247
Median filter	0.328	0.773	0.386	2.992
Hampel filter	0.211	0.838	0.464	3.499
Proposed full framework	0.823	0.397	0.266	1.491

## Data Availability

The original contributions presented in this study are included in the article. Further inquiries can be directed to the corresponding authors.
